# Superhydrophobic Nanosilica Decorated Electrospun Polyethylene Terephthalate Nanofibers for Headspace Solid Phase Microextraction of 16 Organochlorine Pesticides in Environmental Water Samples

**DOI:** 10.3390/polym14173682

**Published:** 2022-09-05

**Authors:** Hamid Najarzadekan, Hassan Sereshti, Irfan Ahmad, Syed Shahabuddin, Hamid Rashidi Nodeh, Nanthini Sridewi

**Affiliations:** 1 School of Chemistry, College of Science, University of Tehran, Tehran 1417614411, Iran; 2 Department of Clinical Laboratory Sciences, College of Applied Medical Sciences, King Khalid University, Abha 61421, Saudi Arabia; 3 Department of Chemistry, School of Technology, Pandit Deendayal Energy University, Raisan 382426, Gujarat, India; 4 Food Technology and Agricultural Products Research Center, Standard Research Institute, Karaj 3174734563, Iran; 5 Department of Maritime Science and Technology, Faculty of Defence Science and Technology, National Defence University of Malaysia, Kuala Lumpur 57000, Malaysia

**Keywords:** electrospun nanofibers, polyethylene terephthalate, superhydrophobic nanosilica, solid-phase microextraction, organochlorine pesticides

## Abstract

A new solid phase micro extraction (SPME) fiber coating composed of electrospun polyethylene terephthalate (PET) nanofibrous mat doped with superhydrophobic nanosilica (SiO_2_) was coated on a stainless-steel wire without the need of a binder. The coating was characterized by scanning electron microscopy (SEM) and Fourier transform infrared spectrometer (FTIR) techniques and it was used in headspace-SPME of 16 organochlorine pesticides in water samples prior to gass chromatography micro electron capture detector (GC-µECD) analysis. The effects of main factors such as adsorption composition, electrospinning flow rate, salt concentration, extraction temperature, extraction time, and desorption conditions were investigated. Under the optimum conditions, the linear dynamic range (8–1000 ng L^−1^, R^2^ > 0.9907), limits of detection (3–80 ng L^−1^), limits of quantification (8–200 ng L^−1^), intra-day and inter-day precisions (at 400 and 1000 ng L^−1^, 1.7–13.8%), and fiber-to-fiber reproducibility (2.4–13.4%) were evaluated. The analysis of spiked tap, sewage, industrial, and mineral water samples for the determination of the analytes resulted in satisfactory relative recoveries (78–120%).

## 1. Introduction

Organochlorine pesticides (OCPs) are persistent lipophilic organic pollutants that are highly resistant to biodegradation in the environment [1]. These compounds exhibit high toxicity and bioaccumulation and have been demonstrated to be carcinogenic in animals and humans. For these reasons, they have been included in the list of priority pollutants compiled by The United States-Environmental Protection Agency (US-EPA) [2]. Although the application of OCPs has been forbidden for a considerable period in many countries, the residues continue to induce a significant impact on the environment and its ecosystems [3]. The maximum possible limits are 0.1 µg L^−1^ for each OCP and 0.5 µg L^−1^ for the total concentration of all pesticides based on toxicological considerations [4,5]. Therefore, it is necessary to develop fast, simple, and valid methods for their determination in different matrices [2]. A variety of sample preparation techniques, such as pressurized liquid extraction [6], in-cell accelerated solvent extraction [7], microsolid-phase extraction [8], magnetic solid-phase extraction (MSPE) [9], vortex-assisted MSPE [10], and SPME [11] coupled with gas chromatography, have been used for the extraction and determination of OCPs in different matrices. Among these methods, SPME was developed in the 1990s by Arthur and Pawliszyn et al. [12], which satisfies the requirements of green analytical chemistry. Hence, this method has widely been applied for sampling a broad spectrum of analytes from gaseous, liquid, and solid media with diverse matrix compositions [13]. Polydimethylsiloxane (PDMS), divinylbenzene (DVB), carboxen (CAR), polyethylene glycol (PEG), carbowax (CW), polyacrylate, and their combinations, such as PDMS/DVB, PDMS/CAR, CW/DVB, and DVB/CAR/PDMS, are the commercially-available SPME fiber coatings [14,15]. However, the ordinary SPME fibers suffer from poor mechanical strength, low recommended operating temperature, fragility, restricted lifetime, and limited applicability [16]. To overcome these drawbacks, researchers have tried to synthesize new sorption materials for SPME purposes. In recent years, the use of nanoscale materials with diverse functionalities and polarities for the fabrication of new SPME fiber coatings with enhanced selectivity, sorption capacity, and stability has attracted the attention of researchers [17,18]. Moreover, nanoscale materials with a high specific surface area can improve the sample loading capacity and extraction efficiency [17,19,20,21]. Therefore, due to the unique features of the nanoscale materials, they have emerged and become important in the analytical detection and remediation of environmental pollutants [22]. Furthermore, in some SPME applications, low-cost metal wires have been replaced with fragile fused silica fibers [23,24]. The electrospinning method, which is a popular technique for the production of polymeric nanofibers [25], has been widely used as an SPME fiber coating [26,27,28,29]. The polymers can be embedded with different materials to enhance the performance of the coating by increasing the surface area-to-volume ratio and the functionality of the produced nanofibers [30,31].

In the present study, an effective head space solid phase microextraction (HS-SPME) adsorbent using electrospun composite nanofibers was used for the extraction of OCPs from aqueous solutions. Accordingly, different PET-based nanofibers, such as PET/nanoclay, PET/nano-SiO_2_, and PET/calixarene, were electrospun on the surface of a stainless-steel wire and tested under the same experimental conditions. To the best of our knowledge, this is the first time that these PET-based composite nanofibers have been fabricated and applied for the extraction of OCPs.

## 2. Experimental

### 2.1. Chemicals and Reagents

The 16 OCP mixture (EPA 608 pesticide mix, a stock standard solution of 20 µg mL^−1^ OCPs in toluene:n-hexane, (1:1)) was purchased from Sigma-Aldrich (St Louis, MO, USA). The mixture consisted of α-hexachlorocyclohexane (HCH), β-HCH, γ-HCH, δ-HCH, aldrin, dieldrin, endosulfan I, endosulfan II, endosulfan sulfate, endrin, endrin aldehyde, heptachlor, heptachlor epoxide-isomer B, p,p′-DDE, p,p′-DDD, and p,p′-DDT. All the standard solutions were diluted with distilled water and acetone and later stored at −4 °C in the dark. Trifluoroacetic acid (TFA, 99%) was supplied from Samchun Pure Chemical (Seoul, South Korea). Polyethylene terephthalate (PET) was bought from Merck Chemicals (Darmstadt, Germany). NaCl (ACS reagent, ≥99.0%, Sigma-Aldrich, St Louis, MO, USA) was used as received. The superhydrophobic nano-SiO_2_ and nano-clay were obtained from Nanosav Company (Tehran, Iran).

### 2.2. Gas Chromatography 

An Agilent Technologies gas chromatograph (6890N, Santa Clara, CA, USA) equipped with a micro electron capture detector (µ-ECD) and an HP-5 capillary fused silica column (30 m × 0.32 mm × 0.25 µm) was used for the separation of the extracted OCPs. Helium (99.999%) at a flow rate of 1 mL min^–1^ and nitrogen (99.999%) at a flow rate of 30 mL min^−1^ were used as the carrier gas and makeup gas, respectively. The injector and detector temperatures were set at 200 and 300 °C, respectively. The sample introduction was performed in the splitless mode for 3 min. The column temperature program was initiated at 60 °C (held for 1 min), increased at 30 °C min^−1^ to 180 °C, and then raised to 250 °C at 7 °C min^−1^ (fixed for 5 min).

### 2.3. Instrumentation

A Zeiss DSM-960 SEM (Oberkochen, Germany) at the accelerating voltage of 20 kV was used for morphology characterization. An Equinox 55 FT-IR spectrometer (Bruker, Bremen, Germany) was utilized for recording the infrared spectra with KBr pallet. The electrospinning device (Fanavaran Nano-Meghyas Co., Tehran, Iran) consisted of a DC high-voltage power supply and a syringe pump, which was applied for the electrospinning of nanofibers. A homemade SPME syringe with two spinal needles—the internal G 27as SPME-coated needle and the external needle of G 22 as an SPME barrel—was used in headspace–SPME.

### 2.4. Electrospinning of Nanofibers

**Figure 1:**
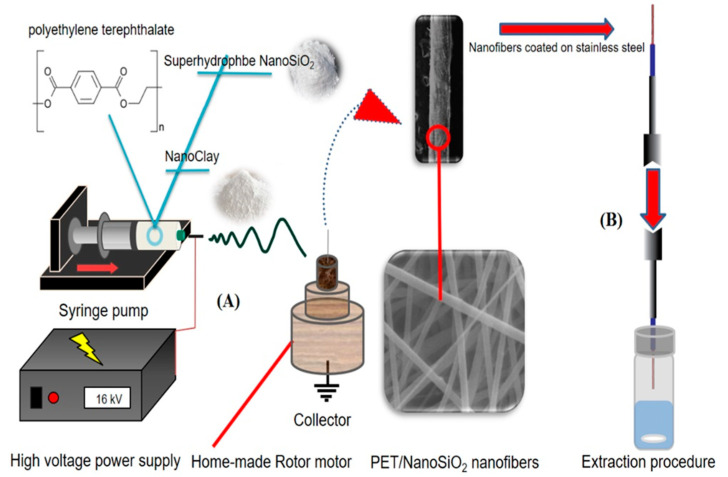
(**A**) The setup design for electrospinning process, and (**B**) headspace–SPME procedure.

The PET/nano-SiO_2_ nanofibers were fabricated as follows. First, 180 mg of polyethylene terephthalate (PET) was dissolved in 1 mL of trifluoroacetic acid (TFA). Then, 5 mg of nano-SiO_2_ was added to the solution and stirred for 100 min to obtain a homogeneous solution. Next, the mixture was transferred into a 2 mL syringe, placed in the syringe pump, and pumped at the rate of 0.15 mL h^−1^. The electrospun nanofibers were collected on the stainless-steel wire attached to a rotating electric motor at a distance of 10 cm from the tip of the syringe’s needle for 8 min (Figure 1A). The electrospinning voltage was 16 kV.

### 2.5. The Procedure 

The fabricated fiber coating was conditioned prior to use by inserting it in the GC injection port at 200 °C for 15 min. Then, 5 mL of the sample solution was placed in a vial, and 1.25 g of NaCl was added to it and stirred for 5 min. After that, the solution was spiked with the standard mixture of the OCPs (100 ng mL^−1^) and sealed by a Polytetrafluoroethylene (PTFE) (CNW, Beijing, China) septum. Next, the needle coated with fibrous PET/nano-SiO_2_ was exposed to the headspace of the sample solution at 40 °C (Figure 1B). Finally, the fiber was withdrawn and immediately inserted into the GC injection port for thermal desorption of the analytes at 200 °C for 3 min.

## 3. Results

### 3.1. Effect of Adsorbent Composition 

**Figure 2:**
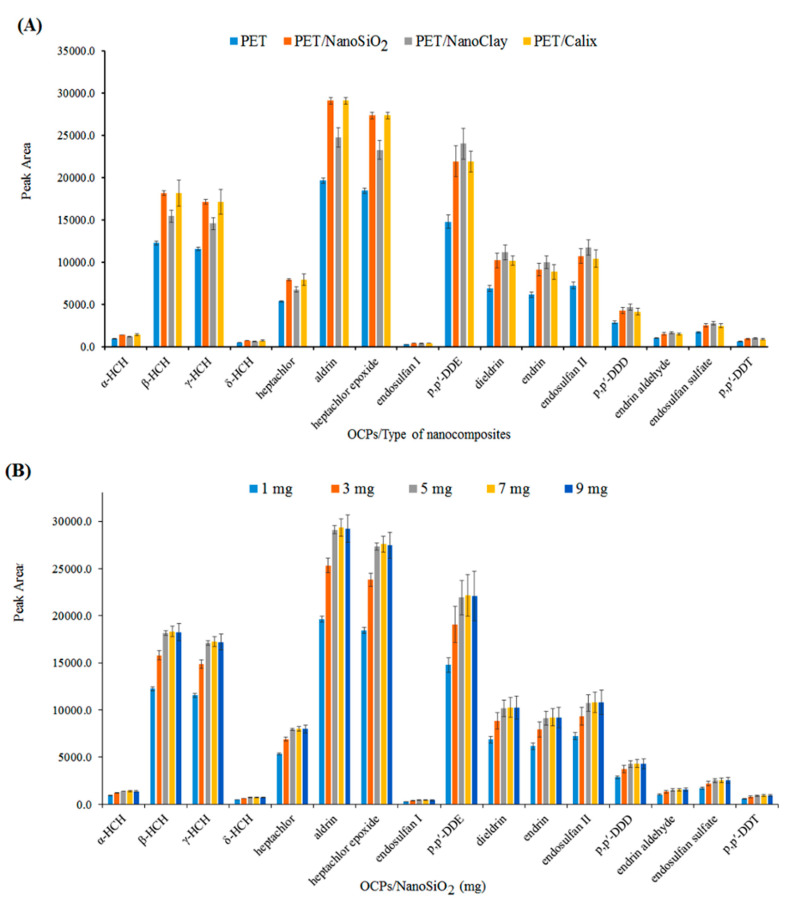

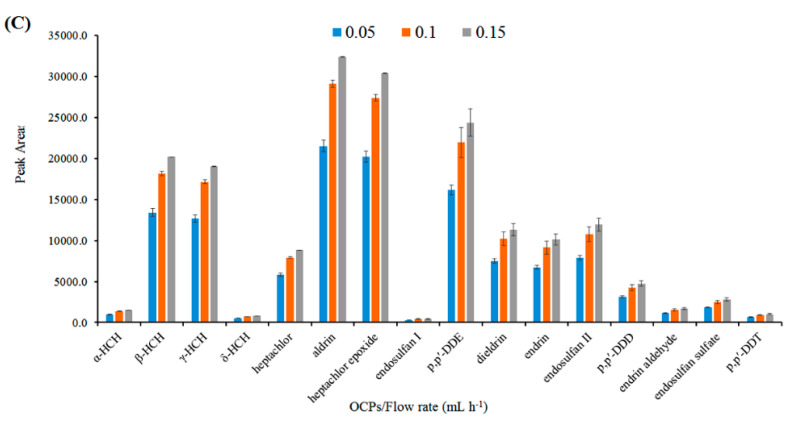
(**A**) Performance of different types of PET nanofibers, (**B**) dosage of nano-SiO_2_ (mg) in PET nanofibers, and (**C**) the effect of polymer solution flow rate (mL h^−1^).

Polyethylene terephthalate was doped with different materials, such as nanoclay, nano-SiO_2_, and calixarene, and electrospun. The nanofibers were tested with the procedure in Section 2.5 and the data was presented in Figure 2A. Clearly, the PET/nano-SiO_2_ had the highest extraction efficiency. Thus, it was selected as the fiber coating for further analyses. In addition, the effect of the nano-SiO_2_ dose in the electrospun nanofibers was also investigated by adding various amounts of nano-SiO_2_ (1−9 mg) into the PET/TFA polymer solution before electrospinning. Figure 2B shows that with an increasing nano-SiO_2_ dose, until 5 mg, the efficiency was increased and remained almost constant after that. Thus, 5 mg was chosen as the optimum amount of nano-SiO_2_.

### 3.2. Effect of Electrospinning Flow Rate

The influence of the electrospinning flow rate on the efficiency of the PET/nano-SiO_2_ nanofibers was studied in the range of 0.05−0.15 mL h^−1^. The higher flow rates were not tested because the thickness of the adsorbent layer was limited by the internal diameter of the SPME needle. As shown in Figure 2C, by increasing the flow rate, the extraction efficiency was also increased. This increase could be due to the higher surface area and porosity of the coating. Therefore, 0.15 mL h^−1^ was selected as the optimum flow rate.

### 3.3. Characterization 

**Figure 3:**
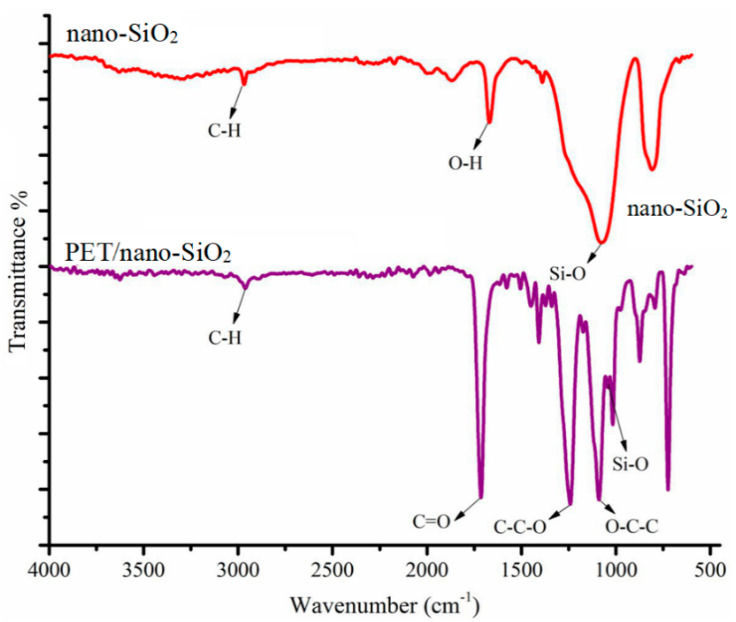
The FT-IR spectra of electrospun nano-SiO_2_ and PET/nano-SiO_2_.

The nano-SiO_2_ and PET/nano-SiO_2_ surface functional groups were studied using FT-IR spectroscopy. The spectrum of nano-SiO_2_ in Figure 3 shows the peaks at 2925, 1631, and 1073 cm^−1^ that can be assigned to the alkane C–H stretching vibrations, O–H bending vibrations, and Si–O stretching vibrations, respectively. Figure 3 presents the FTIR spectrum of PET/nano-SiO_2_, in which the bands at 1728 (C=O), 1243 (C–C–O), 1034 (O–C–C), and 1069 cm^−1^ (Si–O) indicate the successful deposition of nano-SiO_2_ particles on PET [32].

**Figure 4:**
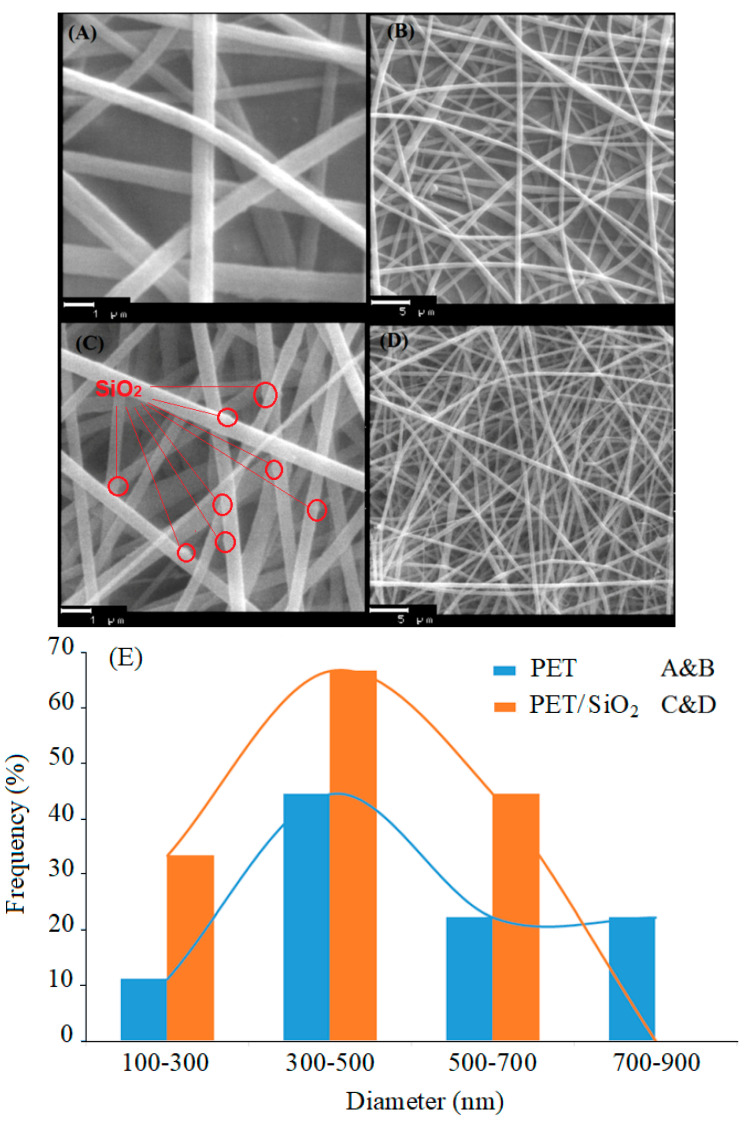
The SEM images of (**A**,**B**) electrospun PET and (**C**,**D**) PET/nano-SiO_2_. (**E**) histogram for size distribution of fabricated nanofiber.

Figure 4A–D shows the SEM micrograph of the PET and PET/nano-SiO_2_ nanofibers in two scales with a histogram of the diameter distribution for nanofibers. These images indicate the three-dimensional porous structure of randomly-oriented fibers with approximately uniform diameters in the ranges of 300–890 nm and 190–615 nm for PET and PET/nano-SiO_2_, respectively. Figure 4E depict the histogram for size distribution of fabricated nanofiber, which is average size obtained 300 to 500 nm.

### 3.4. Effect of Parameters on Extraction Efficiency

#### 3.4.1. Effect of Salt Concentration

**Figure 5:**
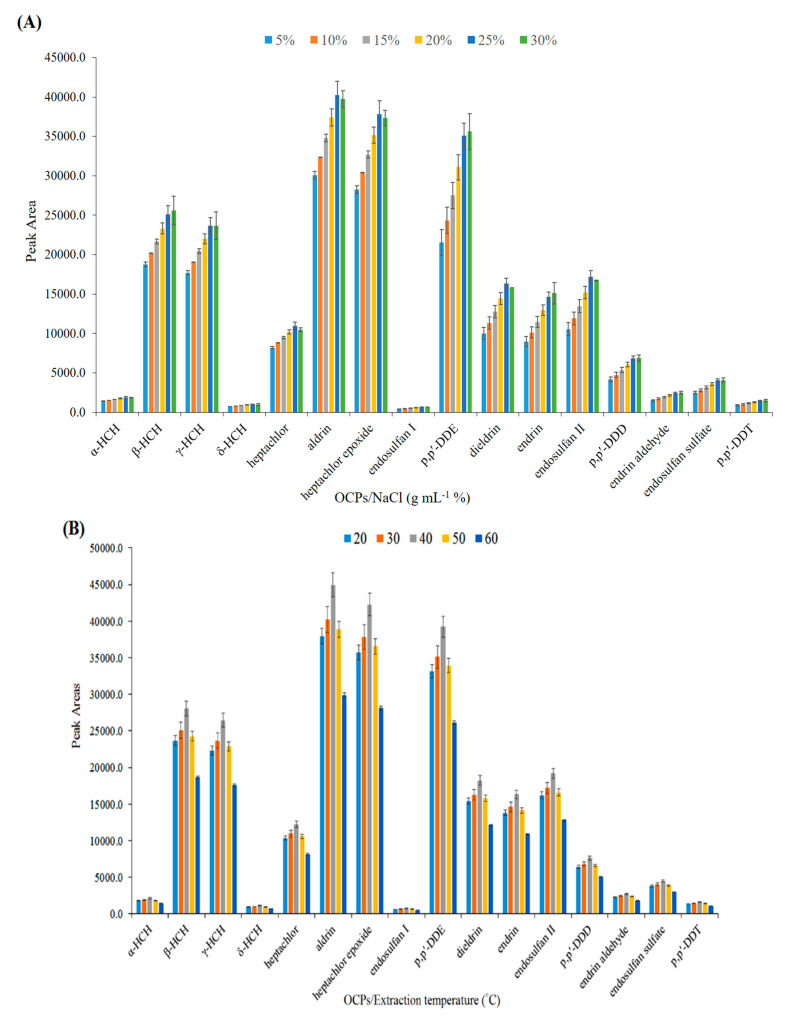

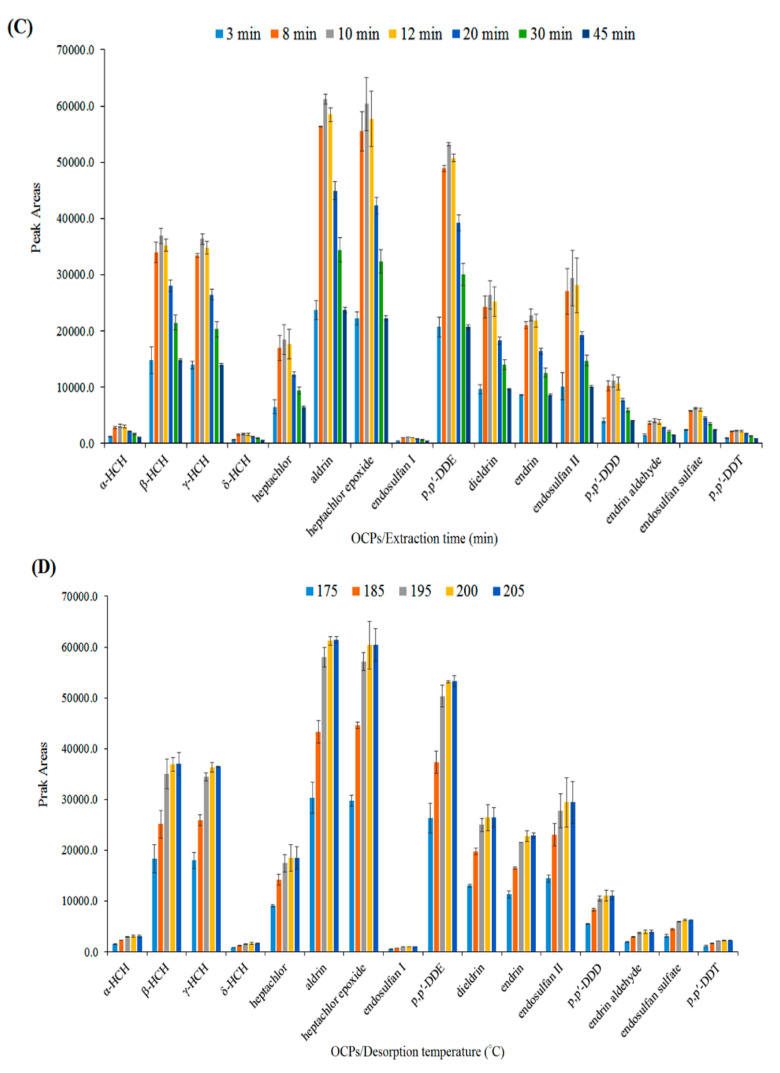

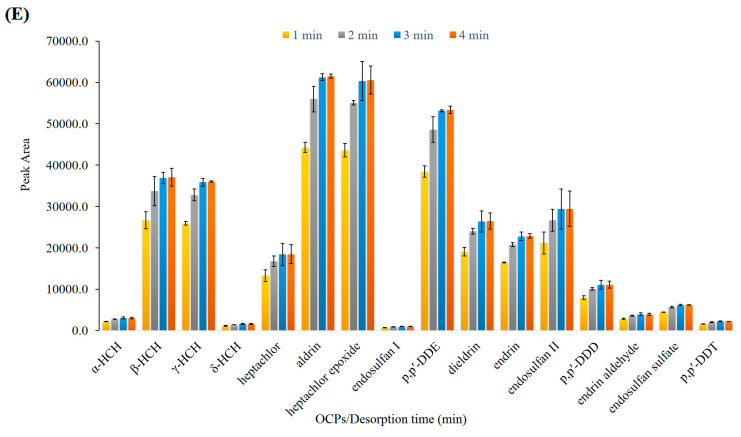
Optimization of effective parameters on extraction efficiency of OCPs. (**A**) Effect of salt (*w*/*v* %), (**B**) extraction temperature (°C), (**C**) extraction time (min), (**D**) desorption temperature (°C), and (**E**) desorption time (min).

The effect of NaCl concentration on the headspace extraction of OCPs was investigated in the range of 5–30% (*w*/*v*). As shown in Figure 5A, the extraction efficiency increased from the addition of salt by up to 25% *w*/*v* due to the salting-out effect. At higher concentrations, the analytical signals remained almost constant, thus 25% was selected as the optimum salt concentration.

#### 3.4.2. Effect of Extraction Temperature

In the HS-SPME technique, the temperature of the sample solution can affect the extraction rate and equilibrium. The increase in temperature accelerates the transfer of the analyte between phases and affects the extraction efficiency through partition coefficients, i.e., *K_hs/s_* and *K_f/hs_* (Equations (1) and (2)), in such a way that improves the former and worsens the latter.

(1) $$K_{hs/s}=\frac{C_{hs}}{C_{os}\;}$$

(2) $$K_{f/hs}=\frac{C_{f}}{C_{hs}\;}$$

where *K_hs/s_*, *K_f/hs_* are the partition coefficients of the analyte in sample/headspace, and headspace/fiber, respectively; and *C_hs_*, *C_s_*, and *C_f_* show the concentrations of analytes in the headspace, sample solution, and nanofibers, respectively. The influence of temperature on the extraction efficiency was studied in the range of 20–60 °C. Figure 5B shows that the extraction efficiency increased from 20–40 °C and declined at higher temperatures. The increase in efficiency in the first region is due to the increase in the concentration of analytes in the headspace, but the decrease in efficiency in the second region can be attributed to the decrease of *K_f/hs_* [13]. Thus, 40 °C was selected as the optimum temperature for the subsequent experiments.

#### 3.4.3. Effect of Extraction Time

The performance of HS-SPME is based on the equilibrium between the adsorbent, headspace, and sample solution. Therefore, the diffusion of the analytes through this triple-phase system is essential. The time taken to reach equilibrium is usually long and the extraction is often involved in non-equilibrium conditions. The influence of extraction time was studied by varying the exposure time of the fiber to the headspace of the sample solution in the range of 3–45 min. Figure 5C shows that the extraction efficiency increased by increasing the extraction time to 10 min, and was thereafter reduced. This short equilibrium time could be attributed to the high surface area and porosity of the nanofibers. Therefore, 10 min was selected as the optimum extraction time.

#### 3.4.4. Effect of Desorption Temperature and Time

The temperature and time of desorption are important factors that influence the efficiency of the SPME process. To ensure the complete transfer of analytes from fiber coating to the GC column, the GC inlet system was operated in the splitless mode. The effect of the desorption temperature (GC inlet) was studied in the range of 175–205 °C. As shown in Figure 5D, the extraction efficiency increased from 175–200 °C and remained almost constant afterward. Thus, 200 °C was chosen as the optimum desorption temperature. Then, the effect of desorption time was investigated in the range of 2–4 min at 200 °C. Therefore, regarding the obtained results in Figure 5E, 3 min was selected as the optimum desorption time to achieve the total desorption of all analytes with no carryover effect.

### 3.5. Method Validation 

Under the optimum conditions (salt concentration, 25% (*w*/*v*); extraction temperature, 40 °C; extraction time, 10 min; and desorption temperature, 200 °C; and desorption time, 3 min), the quantitative performance of the developed method was assessed, and the results were given in Table 1. The calibration curves were prepared using the mixed standard solutions of the Ops at nine concentration levels. The curves were linear in the range of 8–1000 ng L^−1^, with a satisfactory determination coefficient (R^2^) of >0.9971. The limits of detection based on the signal-to-noise ratio (S/N) of three replicates that were 3–80 ng L^−1^. The limits of quantification (S/N, 10) were calculated as 8–200 ng L^−1^. The intra-day relative standard deviations (RSD%, n = 3) at the concentration levels of 400 and 1000 ng L^−1^ were within the 1.7–13.8% range. The inter-day RSD% was calculated using fiber in three different days with three replicates on each day equal to 1.7–11.8% (C = 400 and 1000 ng mL^−1^). The reusability of the PET/nano-SiO_2_ fiber was also evaluated by assessment of its extraction performance in successive adsorption/desorption cycles under the same experimental conditions. After completing each cycle, the adsorbent was washed three times with MeOH and water sequentially. Then, the dried adsorbent was reused for the next run. The results indicated that the fiber can be reused at least 75 times without a significant reduction in efficiency (<5%). Therefore, the PET/nano-SiO_2_ fiber qualified for frequent use in solid-phase extraction-based methods. 

### 3.6. Analysis of Real Samples

Four real water samples including tap water, sewage water, industrial wastewater, and mineral water were selected to assess the applicability of the proposed method for the determination of the selected OCPs. The real samples were spiked with the mixed standard solutions of the target analytes at two concentration levels (400 and 1000 pg mL^−1^). The unspiked and spiked sample solutions were analyzed with the proposed procedure in Section 2.5. The relative recoveries for the spiked samples were calculated by Equation (3), and the results were given in Table 2.

(3) $$RR\left(\%\right)=\frac{C_{found}-C_{real}}{C_{added}}\times 100\;$$

where *C_found_* is the concentration of analytes after spiking the real sample with a standard solution, *C_real_* is the concentration of analytes in the real sample, and *C_added_* is the concentration of standard solution added to the real sample. 

A comparison study was conducted based on the literature survey for the previously reported works for the determination of OCPs, and the data is shown in Table 3. The results indicated that the extraction time of the proposed method is shorter than that of the other methods. In addition, the recovery, linear dynamic range (LDR), and limit of detection (LOD) of the developed method are better than most of the other methods.

## 4. Conclusions

The novel electrospun composite nanofibers of PET superhydrophobic nano-SiO_2_ were fabricated and used as effective fiber coating in HS-SPME. The novel nono fiber was applied for SME extraction of 16 organochlorine pesticides from water samples and thermally desorbed and analysed with GC-µECD. After characterization, the large specific surface area, the porous structure of the adsorbent were obtained. Based on this, the adsorbent and the analytes led to a fast equilibrium (10 min) and efficient extraction with recovery > 90%. Moreover, the low limit of detection (3–80 ng L^–1^) and good linearity (8–1000 ng L^–1^) are the characteristics that provide an excellent sensitivity of the OCPs analytes. Hence, the high efficiency, low LOD and appropriate repeatability are probably due to the large pores structure and effective π-π interactions between the OCPs and PET/SiO_2_. In addition, the method is eco-friendly since it requires no organic solvent in the extraction and analysis steps.

## Figures and Tables

**Table 1:** Analytical data obtained by HS-SPME of OCPs mixture using the PET/nano-SiO_2_ adsorbent.

Compound	LOD ^a^	LOQ ^b^	LDR ^c^	R^2^	RSD ^d^			RSD ^e^		
					Intraday	Interday	Fiber to Fiber	Intraday	Interday	Fiber to Fiber
α-HCH	30	80	80–10,000	0.9967	6.3	8.6	7.5	2.2	6.4	5.4
β-HCH	20	50	50–5000	0.9914	5.3	1.7	3.5	3.1	2.4	2.4
γ-HCH	20	50	50–5000	0.9914	4.8	9.5	7.1	3.2	7.0	6.3
δ-HCH	80	200	200–10,000	0.9983	8.1	10.6	4.8	8.2	8.8	11.9
Heptachlor	20	50	50–5000	0.9907	10.5	7.3	8.9	5.5	6.0	6.4
Aldrin	3	8	8–2000	0.9977	6.2	4.5	5.4	4.0	3.9	4.2
Heptachlor epoxide	3	8	8–2000	0.9930	5.5	2.6	4.0	5.5	2.7	4.0
Endosulfan I	80	200	200–10,000	0.9987	10.9	10.3	11.6	5.7	10.6	12.0
p,p′-DDE	3	8	8–2000	0.9989	7.2	6.5	6.8	2.6	4.2	4.5
Dieldrin	30	80	80–5000	0.9909	11.3	7.6	11.5	4.2	6.0	5.9
Endrin	30	80	80–5000	0.9987	9.8	3.1	6.5	1.7	3.4	2.4
Endosulfan II	30	80	80–5000	0.9932	12.3	8.1	10.2	5.6	4.3	6.9
p,p′-DDD	30	80	80–2000	0.9912	12.4	8.5	5.4	10.0	8.8	9.3
Endrin aldehyde	30	80	80–2000	0.9942	13.8	8.0	5.4	9.5	11.0	12.3
Endosulfan sulfate	30	80	80–5000	0.9921	11.4	9.3	7.3	8.5	6.6	11.4
p,p′-DDT	30	80	80–5000	0.9965	11.3	10.5	11.4	11.3	11.8	13.4

^a^ Limit of detection (ng L^−1^). ^b^ Limit of quantification (ng L^−1^). ^c^ Linear dynamic range (ng L^−1^). ^d^ C = 400 ng L^−1^. ^e^ C = 1000 ng L^−1^.

**Table 2:** Analytical data obtained after HS-SPME of OCPs mixture using the PET/Superhydrophobe NanoSiO_2_.

Compound	Industrial Water ^a^ (RR%) ^b^				Sewage Water ^c^ (RR%)				Tap Water (RR%)				River Water ^d^ (RR%)			
	S1 ^e^	RSD%	S2 ^f^	RSD%	S1	RSD%	S2	RSD%	S1	RSD%	S2	RSD%	S1	RSD%	S2	RSD%
α-HCH	100.3	6	99.6	6.6	93.7	7.8	99.6	6.6	90.8	6.3	99.4	2.2	96.5	11.3	101	4.9
β-HCH	114.9	2.9	97.7	2.4	98	1.5	97.7	2.4	89.8	5.3	99.8	3.1	105.1	2.5	99.3	3.9
γ-HCH	88.8	3.5	93.3	7.7	103.3	1.5	93.3	7.7	91.2	4.8	100.1	3.2	90.3	1.7	96.4	5.0
δ-HCH	95.4	18	95.1	13.6	120.7	7.3	95.1	13.6	103.1	48.1	100.4	8.2	113.2	17.0	98.1	9.8
Heptachlor	98.9	11	97.1	6.9	98.4	10.1	97.1	6.9	100.8	10.5	99.6	5.5	103.1	6.9	100.9	6.3
Aldrin	96.0	1.3	98	4.6	98.2	5.2	98	4.6	102.3	6.2	100	4.0	101.5	4.9	100.5	4.3
Heptachlorepoxide	98.9	5.9	96.7	1.7	101.3	4.4	96.7	1.7	100.1	5.5	99.7	5.5	102.0	3.1	96.0	1.7
Endosulfan I	100	10.4	96.2	13.1	116.1	10.7	96.2	13.1	95.3	10.9	100.5	5.7	102.5	9.2	101.1	5.2
p,p′-DDE	95.2	1.3	99.6	4.2	98.9	7.0	99.6	4.2	96.0	7.2	100.3	2.6	101.5	6.1	102.2	2.2
Dieldrin	87.3	7.0	97.1	6.4	90.9	4.8	97.1	6.4	92.8	21.3	99.4	4.2	105.8	14.7	96.5	5.5
Endrin	103	7.3	100.8	3.7	99.9	4.1	100.8	3.7	101.4	9.8	100.5	1.7	100.3	9.8	99.6	3.0
Endosulfan II	97.9	13.7	96.0	5.1	95.7	10.1	96	5.1	99.7	12.3	100.2	5.6	103.2	8.7	95.8	5.2
p,p′-DDD	88.3	7.0	96.1	11.0	92.6	13.8	96.1	11	90.0	22.4	99.6	10.0	106.1	15.5	96.4	11.5
Endrin aldehyde	81.3	10.1	100	13.2	94.1	12	100	13.2	97.8	23.8	101.3	9.5	104.7	16.6	99.4	12.2
Endosulfan sulfate	82.4	17	98.3	8.0	79.1	14.5	98.3	8.0	98.5	14.4	100.8	8.5	102.3	11.2	99.2	9.6
p,p′-DDT	78.7	7.0	100.6	18	88.4	14.1	100.6	18	93.1	21.3	100.1	11.3	99.9	16.2	97.3	12.8

^a^ Collected from Khoramdasht industrial park. ^b^ Relative recovery. ^c^ Collected from our university campus. ^d^ A river in the north of Iran. ^e^ Spiked with 400 ng mL^−1^. ^f^ Spiked with 1000 ng mL^−1^.

**Table 3:** Comparison of HS-SPME/GC-ECD with other published method for determination of OCPs.

Methods	Sorbent	LOD ^a^	LDR ^b^	Extraction Time (min)	RR% ^c^	Ref
SPME	PET/NanoSiO_2_	3–80	8–10,000	10	78–120	Current study
SPE-GC/ECD	Florisil	400–2000	5–1000	-	77–105	[33]
SMPE	PDMS ^d^/PA ^e^	20–80	50–1000	20	91.4 (average)	[34]
SB-μ-SPE	Hydroxide/graphene	300–1400	1000–200,000	20	84.2–100.2	[8]
ASE ^f^ & SPME	PDMS/PA	0.2–4.9 (ng m^−3^)	50–3000 (ng m^−3^)	40	-	[35]

^a^ Limit of detection (ng L^−1^). ^b^ Linear dynamic range (ng L^−1^). ^c^ Relative Recovery. ^d^ Polydimethylsiloxane. ^e^ Polyacrylate. ^f^ Accelerated solvent extraction.
